# Nutritional Rickets

**DOI:** 10.4274/jcrpe.v2i4.137

**Published:** 2010-11-01

**Authors:** Behzat Özkan

**Affiliations:** 1 Atatürk University, Faculty of Medicine, Department of Pediatric Endocrinology, Erzurum, Turkey; +90 442 231 17 11+90 532 513 22 99bozkan@atauni.edu.trAtatürk University, Faculty of Medicine, Department of Pediatric Endocrinology, Erzurum, Turkey

**Keywords:** Nutritional rickets, Vitamin D, calcium

## Abstract

Nutritional rickets (NR) is still the most common form of growing bone disease despite the efforts of health care providers to reduce the incidence of the disease. Today, it is well known that the etiology of NR ranges from isolated vitamin D deficiency (VDD) to isolated calcium deficiency. In Turkey, almost all NR cases result from VDD. Recent evidence suggests that in addition to its short− or long−term effects on skeletal development, VDD during infancy may predispose the patient to diseases such as diabetes mellitus, cancer and multiple sclerosis. Among the factors responsible for the high prevalence of VDD in developing countries and its resurgence in developed countries is limited sunshine exposure due to individuals’ spending more time indoors (watching television and working on computer) or avoiding sun exposure intentionally for fear of skin cancer. Traditional clothing (covering the entire body except the face and hands) further limits the exposure time to sunlight and, thus, decreases the endogenous synthesis of vitamin D. In Turkey, maternal VDD and exclusive breastfeeding without supplementation were reported to be the most prominent reasons leading to NR. The diagnosis of NR is established by a thorough history and physical examination and confirmed by laboratory evaluation. Recent reports draw attention to the supplemental doses of vitamin D required to achieve a serum 25−hydroxyvitamin D level of at least 20 ng/ml (50 nmol/l) − the serum concentration that is needed to optimize absorption of dietary calcium and to suppress excessive secretion of parathyroid hormone. This type of prevention will also reduce fracture risk as well as prevent long−term negative effect of vitamin D insufficiency.

**Conflict of interest:**None declared.

## INTRODUCTION

Vitamin D deficiency (VDD) is known to be the leading cause of nutritional rickets (NR). Recent publications indicate that dietary calcium (Ca) deficiency before the occurrence of epiphyseal fusion can also have a primary role in the etiology of this metabolic bone disease (1, 2). In Turkey, almost all NR cases result from VDD, whereas in Egypt and Nigeria, Ca insufficiency and/or VDD have been shown to have a role in the etiology of the condition (3).

**Figures 1:**
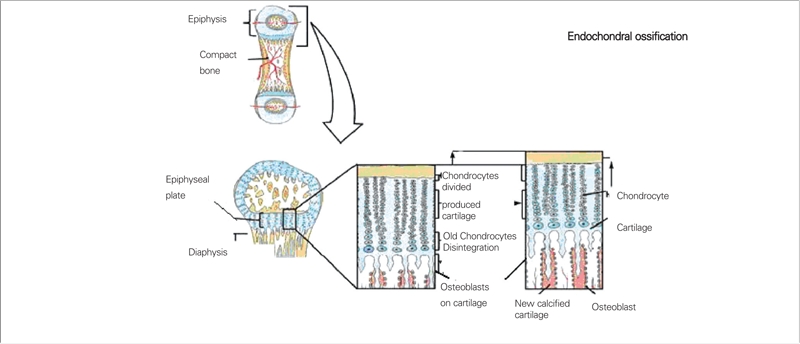
Endochondral ossification of the growth plate

In a vitamin D sufficient state or when the serum 25−hydroxyvitamin D (25(OH)D) level is above 20 ng/mL (50 nmol/L), intestinal Ca absorption can be as high as 80% of the intake, especially during periods of active growth. On the other hand, in a vitamin D deficient state, intestinal Ca absorption can decrease to as low as 10−15% and there is also a decrease in total maximal reabsorption of phosphate. In this state, the low serum ionized Ca^++^ level stimulates parathyroid hormone (PTH) secretion, which leads to release of Ca^++^ and phosphorus (P) from bone in an attempt to maintain normal Ca^++^ levels. Increased PTH levels also lead to increased urinary P excretion. Finally, the decreased levels of serum P and Ca^++^ result in decreased bone mineralization. In addition, the low serum P levels cause failure of the expected apoptosis of hypertrophic chondrocytes and this results in cellular “ballooning“ and disorganization of the growth plate (Figures 1 and 2) (4). Initially, in the developing fetus, prechondrocytes induce the process of bone tissue development with aggregation of mesenchymal cells during the endochondral ossification in the growth plate. This is followed by the formation of chondroblasts, chondrocytes and cartilage matrix.

During ossification of the cartilage tissue, chondrocytes differentiate into sequential morphological cell zones with well−defined margins in the epiphyseal growth plate. These

are known as resting zone, proliferative zone, hypertrophic zone and ossification zone of chondrocytes. The hypertrophic chondrocytes are subject to calcification of the surrounding matrix to form the primary center of ossification before apoptosis. This is followed by vascularization of the calcified tissue and arrival of osteoclasts and osteoblasts to the site. Modelling of bone tissue subsequently takes place. In this way, secondary ossification centers are formed and longitudinal healthy bone growth is ensured until the epiphyses are closed by ossification of the cartilage tissue in the growth plate (2, 3, 4, 5, 6, 7, 8).

In rickets, failure of apoptosis of the hypertrophic chondrocytes results in irregular and deformative expansion of the cartilage tissue formed by hypertrophic chondrocytes in the growth plate (Figure 2). This condition leads to cupping and to a brush−like appearance of the epiphyseal ends on radiograms (Figure 3). The nonoccurrence of apoptosis of hypertrophic chondrocytes is reported to be correlated with hypophosphatemia and leads to enlarged unmineralized osteoid tissue seen in the growth plate (8).

**2:**
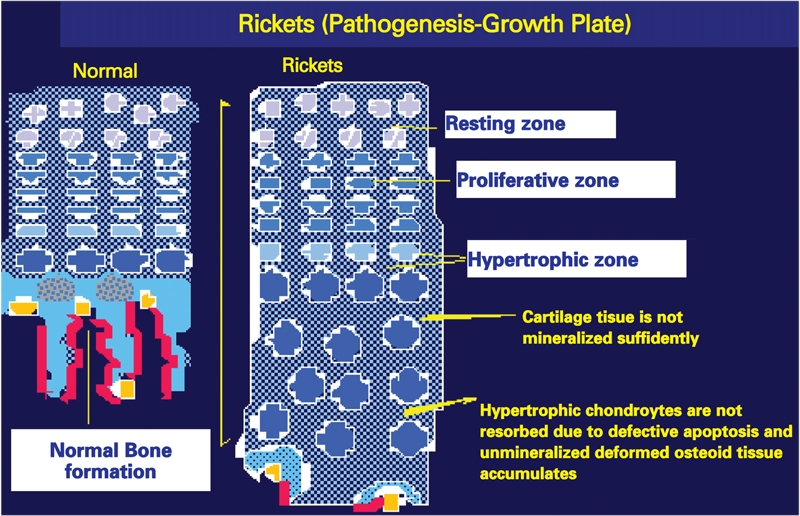
In rickets, the growth cartilage formed by hypertrophic chondrocytes is not resorbed due to the defective apoptosis and the irregularly calcified growth plate expands

**Figure 3:**
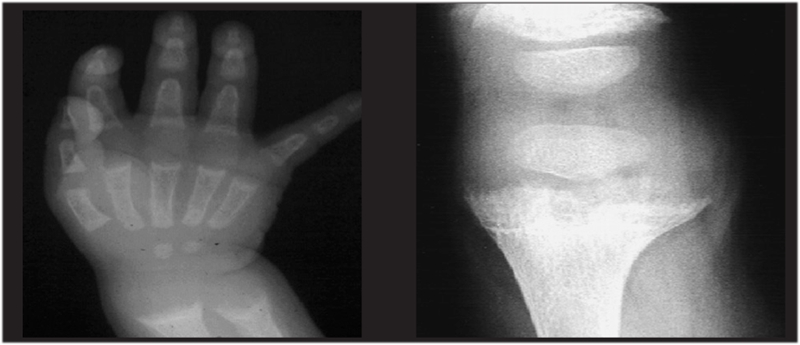
Radiological appearance of cupping and brush−like structure of epiphyses in rickets

## PREVALENCE

Data compiled from various studies reveal that the prevalence of vitamin D insufficiency in Turkey, particularly in fertile women of reproductive age, is as high as 80% (7, 9, 10, 11, 12). This condition increases the incidence of VDD/insufficiency during early infancy (11, 12). The incidence of rickets associated with VDD in infants aged 0−3 years was found to be 6% in a study conducted in the early 2000s in the Eastern Anatolian Region of Turkey, where there is a high incidence of rickets (13). VDD or insufficiency is reported to be a common problem among adolescents also in developed countries. In the United Kingdom, adolescents were found to have the highest rate of VDD among the young population, with over 40% having 25(OH)D levels lower than 20 ng/mL. Seventy percent of adolescent boys in France were found to have 25(OH)D levels less than 10 ng/mL during winter. It would be assumed that in the sunniest areas of the world, this problem would not be common. However, in studies from Saudi Arabia, Iran, Australia, India, Brazil, Lebanon and Turkey, 30−50 % of children and adults were reported to have levels of vitamin D less than 20 ng/mL (14, 15, 16, 17). In a study from Turkey, the prevalence of vitamin D insufficiency in adolescents was estimated as 72% (18).

In 2005, the Turkish Ministry of Health initiated a campaign of free vitamin D for every infant (19). Following this intervention measure, the incidence of NR in the age group 0−3 years was found to be less than 1% in a research carried out in the Eastern Turkey, where a high frequency of NR was known to exist between 2007 and 2008 (20). These findings indicate that rickets associated with VDD has decreased substantially in Turkey.

## ETIOLOGY

Today, most cases of rickets in developed countries are reported to be in infants exclusively breastfed and born to black mothers or mothers with dark skin and without sufficient storage of vitamin D; spending most of their lives in cities or houses with air pollution; residing at latitudes distant from the equator (>400 north or south latitude) in winter months and without sufficient exposure to sun (Table 1).

The source of vitamin D during fetal life and in the postnatal period is through placental passage, mother’s milk and synthesis in the skin by way of sunlight. Vitamin D levels in infants correlate with vitamin D levels in their mothers during the first 2 months of life. Diet and sunlight determine the levels in the later months and years of life. Based on these facts, the insufficiency of vitamin D storage in mothers as well as exclusive breastfeeding without vitamin D supplementation constitute important risks for NR in early life (4, 7, 11).

Apart from the etiologic factors listed in Table 1, presence of factors such as faulty nutrition of the mother and infant including low vitamin intake, growth and developmental retardation, chronic systemic diseases with a potential impact on the vitamin D metabolism, chronic medication intake (anticonvulsants, glucocorticoids), should be investigated. In cases of rickets, the history should include information on gestational age, extent of exposure to sunlight, and area and geography of residence. A history of

low stature in the family, alopecia, dental deformities, orthopaedic abnormalities, and consanguineous marriage should be investigated for differential diagnosis. Growth, orthopaedic problems, and hypoxemia−related symptoms and findings (muscle cramps, paresthesias, tetany and convulsions) should especially be questioned (1, 2, 4, 7, 12). Hypocalcemia resulting from maternal VDD should also be considered in the differential diagnosis of late neonatal convulsions (21).

**Table 1:**
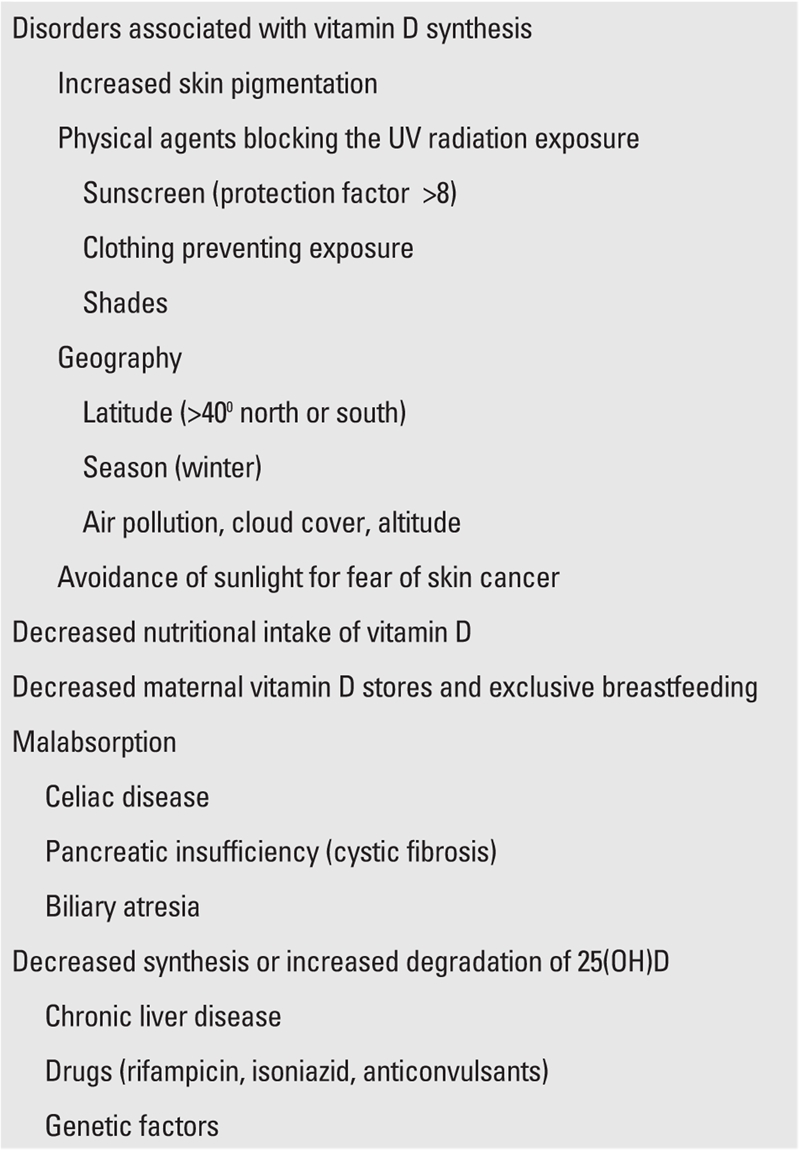
Causes of vitamin D deficiency (VDD)

## PHYSICAL EXAMINATION

Rickets is the disease of a growing organism; therefore, the deformities and clinical findings are more specific to the bone tissue that is undergoing rapid growth at the age of onset of rickets. The growth rate decreases in cases of malnutrition and hypothyroidism, leading to less definite clinical findings. The clinical presentation of NR is stage−dependent and most likely due to the duration of VDD. Hypocalcemic symptoms are predominant in stage I. Skeletal deformities become obvious in stage II and worsen in stage III. Clinical findings of rickets comprise those that are specific to and those that are not specific to the bone tissue. Accordingly, if there is suspicion of rickets, a complete physical and dental examination should be carried out, and the entire skeletal system should be palpated to identify possible sensitivity and deformities.

Physical examination findings specific to the bone tissue in rickets include craniotabes in infants older than 2−3 months, delayed fontanel closure, enlargement of wrists, rachitic rosary, delayed teething, carious teeth, enamel hypoplasia, “O“− or “X“−type leg deformity, kyphosis and a narrow pelvis that may affect labor in later years, chest deformities such as Harrison’s groove and pigeon breast, costal or lower extremity fractures (particularly greenstick fractures), caput quadratum, frontal bossing, brown tumour and extremity pain (1, 11, 14). Deformities caused by softening of the bones of the lower extremities (tibial and femoral) develop once the infant starts walking. Genu varum deformity occurs when the femoral intercondylar distance exceeds 5 cm and it is the most common skeletal system deformity in infants with untreated rickets. Genu valgum and other leg deformities can be expected to develop at later ages. Rickets−related kyphoscoliosis is observed after 2 years of age. Rachitic rosaries caused by hypertrophy in the costochondral junction and palpated as rosary beads are clearly observed after 1 year of age (1, 2).

Physical examination findings not specific to the bone tissue include hypocalcemic convulsions, hypotonia, constipation, proximal myopathy, heart failure, anemia, pancytopenia, cardiomyopathy, benign intracranial hypertension, growth retardation and low height−for−age (1, 2, 11). In a recent study, a 10−month−old breastfed infant with rickets associated myelofibrosis presenting with anemia and hepatosplenomegaly was reported. In this patient, treatment with vitamin D led to a reduction in liver and spleen size along with improvement of rickets, anemia, growth and development parameters (22). NR should also be considered as an important curable cause of dilated cardiomyopathy among children, especially in regions where NR is still common (23).

Thacher et al (24) conducted a study including 736 (>18 months) cases of rickets and concluded that of all clinical signs, the combined finding of enlargement of the wrists and rachitic rosaries constituted the most sensitive physical examination finding. In another study, it was reported that rachitic rosary (62.1%), craniotabes (49%), occipital alopecia (31.4%) and enlargement of the wrists (27.1%) were the four most common physical examination findings for the age group 0−6 months (7). In this study, the positive predictive value of physical examination in absolute diagnosis of rickets for the age group 0−6 months was reported to be 60.9% and the negative predictive value was 74.6%. Thus, diagnosing rickets based solely on the physical examination findings during early infancy may be misleading (7, 12).

## LABORATORY FINDINGS

In a patient suspected to have rickets based on clinical findings, the diagnosis is confirmed by biochemical and radiological findings. Table 2 demonstrates the limit values for VDD and insufficiency for rickets as reported by the Drug and Therapeutics Committee of the Lawson Wilkins Pediatric Endocrine Society (4). According to this Committee, severe deficiency is defined as a 25(OH)D level of lower than 5 ng/mL. In one study, it was reported that 86% of the children studied who had 25(OH)D levels below 8 ng/mL had clinical rickets, and 94% of the hypocalcemic children with VDD had levels below 8 ng/mL (25). In VDD rickets, a certain period of time, varying among different individuals, should elapse before the clinical and radiological findings appear. During this period of time, hypo−normo−hypercalcemia, high levels of PTH, normo/hypophosphatemia, high levels of alkaline phosphatase (ALP), high−normal−low levels of 1,25(OH)2D may be detected, moreover, subclinical rickets will progress to clinical rickets (Stage I−III rickets). 60% of VDD associated rickets cases have hypocalcemia and ALP is high in nearly all cases. Ca/P levels are low and PTH and ALP levels are high, particularly in stage III NR cases (4, 6, 7, 9, 12). Since most rickets cases in early infancy are stage I, clinical and X−ray findings cannot be overt. In addition, although hypocalcemia is the main laboratory finding in these cases, serum P levels can be normal or high and ALP levels can be in the normal range (12).

**Table 2:**
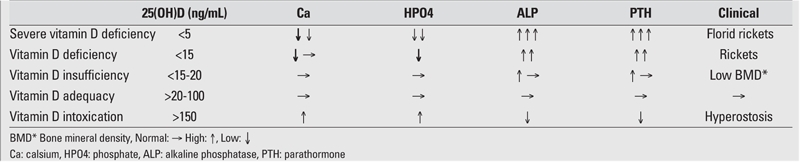
Laboratory findings (serum levels) in NR

## RADIOLOGICAL FINDINGS

The earliest radiological findings are limited to the distal ulnar region in infants and to the lower and upper metaphyses of the knees in older children (1). Initially, a “radiolucent” line resulting from accumulation of the noncalcified metaphysis between the epiphysis and metaphysis is observed. Expansion of the metaphysis, irregularity of the metaphyseal margin, a brush−like appearance, cupping and general osteopenia are the typical radiological findings in classical cases (Figure 3). On the other hand, definite radiological findings may not be present during early infancy and adolescence (7, 26, 27).

A scoring system developed by Thacher (26) is used in the radiological evaluation to establish the severity of rickets (26, 27). However, using the Thacher’s scoring during early infancy may not be a practical approach (7). Bending, fractures of the long bones resulting from thinning of the cortex due to general osteopenia, and expansions in the costochondral junctions (rachitic rosaries) are other radiological findings in cases of rickets (Figure 3, 4). In the first 3−4 weeks following start of treatment, it is possible to radiologically detect the provisional calcification line in the metaphyseal ends in order to identify whether the rickets is improving (1, 28, 29, 30).

**Figure 3:**
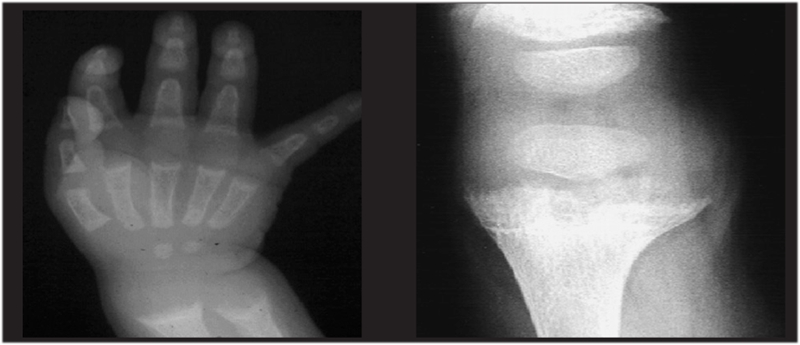
Radiological appearance of cupping and brush−like structure of epiphyses in rickets

**4:**
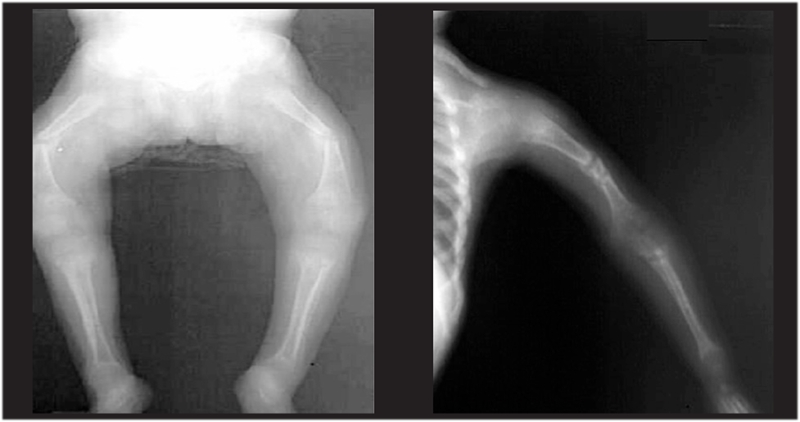
Radiological findings in nutritional rickets (NR)

## TREATMENT

The main objective of treatment is to correct the clinical, biochemical and radiological findings and to restore vitamin D reserve. To this end, inactive vitamin D (cholecalciferol or ergocalciferol) is used. Generally, one of the two treatment methods are preferred.

**Low dosage and long−term vitamin D therapy**: There are different views about the dose and duration of vitamin D therapy. In this treatment model, depending on the age of the c h i l d, vitamin D is usually administered at a dose of 1000− 10 000 IU/day for 2−3 months. In this regimen, vitamin D can be given according to the infant’s age as follows: 1000 IU/day for infants under 1 month of age, 1000 to 5000 IU/ day for children 1 to 12 months old, and 5000 IU/day for children older than 12 months. Afterwards, it is recommended to give maintenance therapy of 400 IU/ day. Levels of Ca and P are normalized in 6−10 days by this therapy, while it takes 1−2 months for PTH to reach normal levels. Depending on the severity of the disease, it may take 3 months for the normal serum ALP levels to be restored and the radiological findings of rickets to disappear. In this treatment model, lack of compliance is an important cause of lack of response (4, 14, 28, 29, 30, 31, 32).

**Stoss therapy**: For patients who are suspected to have poor compliance, a high dose of vitamin D can be given orally or intramuscularly as a single dose of 100 000−600 000 IU after the first month of life (4, 14, 30, 31). Administration of 600 000 units of vitamin D in infantile rickets has been reported to cause hypercalcemia (30). Cesur et al (31) reported that 150 000−300 000 units of vitamin D is an effective and safe method of treatment. A recent study also demonstrated that intramuscular administration of a single dose of 300 000 IU of vitamin D is effective in cases of malnutrition with rickets (33). Shah and Finberg have successfully administrated 100 000 IU of vitamin D every two hours over a twelve−hour period (4, 34). This treatment evokes a rapid clinical response, resulting in biochemical recovery in a few days and radiological recovery in 10−15 days.

Although controversial, some authors recommend Ca therapy for 1−2 weeks to provide elementary Ca also in patients who do not have symptoms of hypocalcemia. Parenteral Ca is usually given as Ca gluconate (1−2 ml/kg of 10% Ca gluconate, providing 10−20 mg/kg of elemental Ca) and is administered intravenously and slowly, over 5−10 minutes. Ca administration becomes necessary when clinical signs of tetany or convulsions are present. Ca levels should then be maintained with oral Ca supplements (4, 14).

## PREVENTION

The most physiological method to prevent vitamin D insufficiency/deficiency is to educate society, and thus, to ensure that mothers and infants are sufficiently exposed to sunlight and eat a balanced diet rich in Ca and vitamin D. Tables 3 and 4 demonstrate foods rich in vitamin D and Ca and the amounts of vitamin D and Ca per portion. Then again, detection of the maternal factors causing vitamin D insufficiency and taking measures to target these factors is essential in preventing cases of early rickets.

For infants, it was initially recommended in the USA to give 100 IU vitamin D per day for the prevention of rickets. In 1963, the American Academy of Pediatrics (AAP) introduced the protocol of 400 IU/day of vitamin D starting from the 2nd month of life for rickets prophylaxis (29). The latest recommendations by AAP about vitamin D supplementation was reported in 2008 (4) as follows:

• A minimum of 400 IU/day vitamin D supplementation is recommended to prevent VDD and rickets in healthy infants, children and adolescents.

• 400 IU/day vitamin D should be introduced to the diet of infants fed completely or partially by mother’s milk until they start receiving at least 1 litre of formula per day.

• 400 IU/day vitamin D should be provided to all infants fed with less than 1 litre formula per day and not receiving mother’s milk. Other sources of nutrition should be calculated individually for infants receiving this type of nutrition.

• 400 IU vitamin D should be provided to all adolescents not receiving 400 IU vitamin D from milk or other foods fortified with vitamin D.

• According to recent evidence, the level of serum 25(OH)D should be above 20 ng/mL, particularly in infants and children.

• 400 IU/day vitamin D should be continued in cases with chronic fat malabsorption, chronic anticonvulsant intake or similar conditions increasing the risk of VDD. Higher vitamin D supplementation may be necessary in these cases to maintain the normal serum level of vitamin D.

The Bone Health Group in Turkey recommends vitamin D supplementation of at least 400 IU/day to be introduced at birth and continued until sufficient vitamin D is provided by the diet. However, it is argued that this dose will prevent the development of clinical rickets, but will fail to prevent vitamin D insufficiency. Therefore, different doses of vitamin D, depending on the risk level, need to be administered for the prophylaxis of VDD. Accordingly, the dose of prophylactic vitamin D is 800 IU/day during winter months in Canada, whereas the dose is 400 IU/day in summer. The dose is 800 IU/day all year round in Bulgaria and 400 IU/day all year round in Romania (29, 33, 34, 35, 36). The Turkish Ministry of Health encourages vitamin D to be administered at a minimum dose of 400 IU/day until 1 year of age for all infants starting from birth, regardless of type of nutrition (9).

Today, vitamin D prophylaxis means not only prevention of clinical rickets (VDD) but also maintenance of optimal serum 25 (OH)D level in order to prevent vitamin D insufficiency. This is an important measure in achieving peak bone mass and even more so in preventing the ill effects of VDD, i.e. diseases such as diabetes and some cancers. It has been reported that the dose of prophylactic vitamin D should be between 400 and 1000 IU/day to maintain the serum 25(OH)D at optimum levels (28−32 ng/mL). Also with a perspective to prevent early rickets, it is recommended that vitamin D at a dose of 2000 IU/day should be administered during the last trimester of pregnancy to mothers with poor exposure to sunlight due to various reasons and who are at high risk of VDD. Various studies have shown that vitamin D up to 2000 IU per day does not cause vitamin D intoxication in adult or child age groups (27).

In conclusion, the vitamin D requirement of a growing child has not clearly been identified. However, we know that children need more vitamin D than the current amounts provided (36). It is up to mothers to supply their children with more Ca−rich foods and sunlight in order to ensure that the vitamin D and Ca requirement of both themselves and their infants is met by natural means.

**Tables 3:**
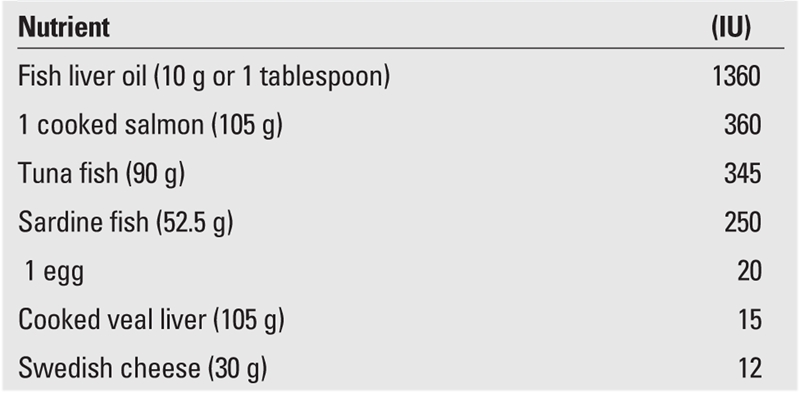
Vitamin D content of various nutrients

**4:**
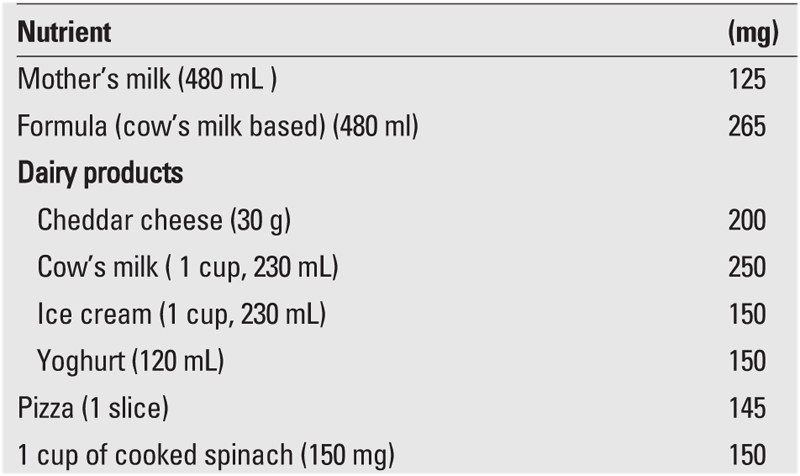
Calsium content of various nutrients
